# Synthesis and Characterization of the Highly Unstable Metalloid Cluster Ag_64_(P^n^Bu_3_)_16_Cl_6_

**DOI:** 10.1002/anie.202006454

**Published:** 2020-07-01

**Authors:** Maximilian Diecke, Claudio Schrenk, Andreas Schnepf

**Affiliations:** ^1^ Chemistry Department University Tübingen Auf der Morgenstelle 18 72076 Tübingen Germany

**Keywords:** jellium model, metalloid clusters, sensitive compounds, silver

## Abstract

The reduction of (^n^Bu_3_P)AgCl with LiBH(^s^Bu)_3_ in toluene gives the metalloid silver cluster Ag_64_(P^n^Bu_3_)_16_Cl_6_ (**1**) as dark red, temperature‐ and light‐sensitive single crystals in high yield. **1** is the largest structurally characterized metalloid silver cluster exhibiting chlorine and phosphine substituents only. The silver atoms in **1** show an overall brick‐shape arrangement, where structural resemblance to the close‐packed fcc and hcp structures is realized. Within **1** a 58 electron closed shell system is present. The light sensitivity renders **1** as a model compound for the primary seeds of the photo process, whereby this sensitivity, together with the high‐yield synthesis show that **1** is a perfect starting compound for further investigations like silver‐plating processes.

## Graphical abstract

**Light up the dark**! The reduction of (^n^Bu_3_P)AgCl with LiBH(^*s*^Bu)_3_ in toluene gives the highly unstable metalloid silver cluster Ag_64_(P^n^Bu_3_)_16_Cl_6_ as dark red, temperature‐ and light‐sensitive single crystals in high yield. This is the largest structurally characterized metalloid silver cluster exhibiting chlorine and phosphine substituents only.

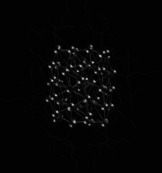

The photographic process has been the bread‐and‐butter work of photographers since the end of the 19^th^ century and thus for more than 130 years. The effect of light‐sensitive substances, like silver halides, forming latent images under light exposure on a medium has been well‐known for many years. Even the chemistry of the photographic processing and developing of a negative image and conversion into a positive image has been in use for a long time and has also been commercialized. Although in the last couple of years the replacement of classical silver‐based photography by computer‐assisted digital images proceeds, the nature of those basic processes of forming latent images on photographic plates is still of fundamental interest. A closer look at the reactions leading to the generation of the latent image shows that the formation of micro‐ or nanoscaled silver particles from silver halide under light exposure is assumed. However, the exact nature of these intermediary formed primary silver nanoparticles is unknown.^1^

Metalloid clusters with a metallic core surrounded by some stabilizing ligands and substituents awakened interest in recent years as small, but crystallizable molecules to provide insight into this area of nanoscaled metal particles.^2^ Metalloid clusters of the general formula M_*n*_R_*m*_ (*n*>*m*, R: organic substituent like Si(SiMe_3_)_3_ or ligand like PPh_3_), which are actually often called “metal clusters” or “metal nanoclusters”,^3^ are ideal model compounds to understand the chemistry of the dissolution or deposition of metals (e.g. Au, Ag, Ga, Al, Sn)^4^ from molecular precursors. Depending on the metal used, metalloid clusters with a size of more than 100 metal atoms in the cluster core are accessible, showing in a few cases similarities to the bulk metal structure, like cubic closed packing (fcc) for all coinage metals.^5^ Furthermore, examples of metalloid clusters of polymorphic metals with more than one solid‐state structure often show structural similarities to different solid‐state structures within their cores; for example, within [Ga_84_(N(SiMe_3_)_2_)_20_]^4−^ a structural relation to α‐Ga and δ‐Ga,^6^ and within Ga_12_(C_13_H_9_)_10_ a structural relation to δ‐Ga^7^ is found. Metalloid clusters might be seen as model compounds for phase‐transition processes as well, like Sn_10_[Si(SiMe_3_)_3_]_6_ (α‐ to β‐tin phase transition).^8^

Many examples of metalloid clusters of the heaviest coinage metal, gold, have been presented in the last years,^9^ but metalloid silver clusters are still quite rare.^10^ The synthesis of [Ag_44_(*p*‐MBA)_30_]^4−^ (*p*‐MBA=*para*‐mercaptobenzoic acid) by Bigioni and co‐workers on a gram scale^11^ was a milestone in this field, where yields of only a few crystals are often the case. In recent years a variety of metalloid silver clusters could be structurally characterized and with [Ag_136_(S‐TBBT)_64_Cl_3_Ag_0.45_]^−^,^12^ Ag_206_(S‐Cyc)_70−*x*_F_*x*_Cl_2_ (*x=*1–4),^13^ and even Ag_374_(S‐TBBT)_113_Br_2_Cl_2_ 12 (TBBT=4‐*tert*‐butylbenzene‐thiolate, Cyc=cyclohexyl) really huge clusters were found. All of these metalloid silver clusters exhibit organic thiolate substituents that form a silver thiolate like shell, showing no light sensitivity. Very recently Wang and co‐workers presented an alkynyl‐proctected metalloid silver cluster, [Ag_112_Cl_6_R_51_]^3−^ (R=CC(3,5‐(CF_3_)_2_C_6_H_3_), which also exhibits halide atoms bound to the silver core;^14^ however, light sensitivity was not reported. In the following, we present the first metalloid silver halide cluster Ag_64_(P^n^Bu_3_)_16_Cl_6_ (**1**), obtained from the reduction of ^n^Bu_3_PAgCl, which can be described as a distorted section of face‐centered cubic packing, showing a high sensitivity to light and even temperature to form metallic silver.

In comparison to the heavier analogue Au, the availability of soluble Ag^I^ precursors is limited due to the very low solubility of Ag^I^X (X=F, Cl, Br, I). However, in the literature a simple synthesis of donor‐stabilized silver halides of the general formula [(R_3_P)_*x*_AgCl]_*y*_ (R: alkyl, *x*,*y=*1–4 depending on R) can be found, which are well soluble, at least in aromatic solvents like benzene and toluene.^15^, ^16^ These Ag^I^ compounds thus seemed to be ideal precursors for the synthesis of metalloid silver clusters via reduction. The reduction of the phosphine‐stabilized silver halides (R=Et, ^n^Pr, ^n^Bu), leads in case of ^n^Bu_3_PAgCl, when reduced with LiBH(*sec*‐Bu)_3_, to a dark red solution, which yields red crystals of the metalloid cluster Ag_64_(P^n^Bu_3_)_16_Cl_6_ **1** after a few days. The extreme light‐ and temperature‐sensitive red single crystals of **1** were prepared for the X‐ray diffraction measurement as quickly and with as little exposure to light as possible, selected in cooled mineral oil (approx. −30 °C) under a dimmed light microscope to prevent decomposition to metallic silver.^17^ Therefore, many attempts were necessary to find a suitable single crystal. This crystal was unfortunately twinned, but the molecular structure of **1** could be refined properly (Figure 1).^18^

**Figure 1:**
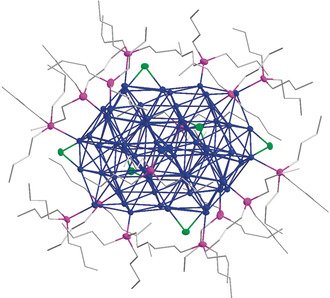
Molecular structure of Ag_64_(P^n^Bu_3_)_16_Cl_6_ **1** in the solid state. H atoms are omitted for clarity. Ag, P, and Cl atoms are shown as thermal ellipsoid with 50 % probability. Bond lengths and angles are given in the Supporting Information. Ag: blue; P: violet, Cl: green, C: gray wires.

The metalloid Ag_64_ cluster **1** crystallized in the triclinic space group *P* $\bar{1}$ with half a molecule in the asymmetric unit. The arrangement of the silver atoms can be described on first glance as an unusual brick‐shaped section of the bulk metal with a distorted fcc arrangement. Usually most of the metalloid clusters can be described by a spherical model, forming characteristic polyhedra. Only a few examples are known where a brick‐like cuboidal arrangement is found that can be also described as a section of the bulk metal, for example, the gold cluster Au_92_(S‐TBBT)_44_ 19 by Jin and co‐workers or Bakr's metalloid silver cluster [Ag_67_(SPhMe_2_)_32_(PPh_3_)_8_]^3+^.^20^

Taking a closer look at the structure of **1**, large differences in the measured Ag–Ag distances, from 280 pm to 335 pm (bulk: 289 pm) are found, already indicating that the arrangement of the silver atoms cannot be described as a simple section of the fcc packing. Within **1**, a central Ag_6_ octahedron is present to which other Ag_6_ octahedra are bound. Thereby edge‐ and face‐sharing of the Ag_6_ octahedra is realized, as highlighted by gray and yellow polyhedra in Figure 2 a, respectively. However, face‐sharing octahedra are not found within an fcc packing but within a hexagonal close packing (Figure 2 b). Hence, within **1** there are areas that are structurally similar to both close‐packed structures (fcc and hcp), which might be induced by the arrangement of the stabilizing ligand shell that consists of 16 P^*n*^Bu_3_ ligands and six chlorine atoms.

**Figure 2:**
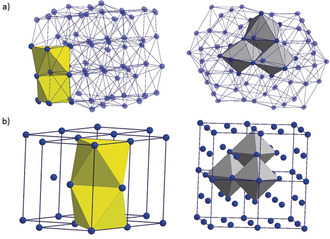
a) Selected polyhedral representation of the Ag core of **1** showing the connection of Ag_6_ octahedra. Gray: edge‐sharing fcc‐like octahedra, yellow: face‐sharing hcp‐like octahedra. b) Octahedral connection mode in ideal hcp (left) and fcc (right) structures.

Although the arrangement of the silver atoms within **1** seems to be distorted with respect to an ideal metallic arrangement, the observed structure seems to be favorable as **1** is obtained in high yield at low temperature in the dark or under red light. The stability of **1** might have electronic grounds as within **1** an electronic shell closure is realized with respect to the jellium model. Hence, within **1**, 58 electrons from the 58 Ag^0^ atoms are available for the silver core. Since 58 is a jellium number (1S^2^ 1P^6^ 1D^10^ 2S^2^ 1F^14^ 2P^6^ 1G^18^),^21^ cluster **1** exhibits a stable closed shell, which might be the reason for the high yield of the synthesis. Although 58 is a so‐called “magic number”, the special structure of the Ag core as a brick‐like arrangement leads to a splitting of the superatomic orbitals, since rather than a spherical a lower symmetric brick‐like background potential is present.^22^ Quantum chemical calculations on **1** reveal that the highest occupied orbitals can indeed be described as superatomic orbitals, as shown in Figure 3: HOMO (p‐like symmetry) and LUMO (g‐like symmetry).^23^

**Figure 3:**
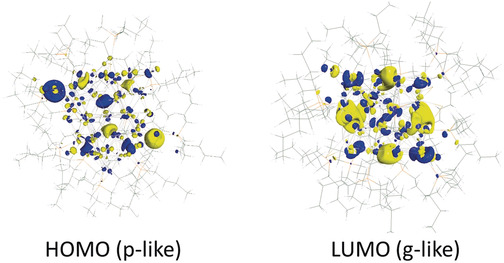
Illustration of the HOMO and LUMO of **1**.^28^

Nevertheless, although **1** is obtained in high yield we found during the various syntheses and attempts at further characterization that **1** is not only sensitive to light, but also quite sensitive to temperature, both in solution and in the solid state. Consequently, the observed temperature sensitivity of the single crystals during X‐ray structure analysis is not or maybe not only due to co‐crystallized solvent molecules, as it is often the case for metalloid clusters. Hence, when single crystals are isolated from the mother liquor under red light at low temperature, dark red crystals are obtained. When those crystals are stored in the dark at room temperature for several days, they become darker (Figure S5 in the Supporting Information); this does not take place at temperatures below −30 °C where the crystals can be stored without decomposition.^24^ Due to this sensitivity further characterization of **1** via NMR or UV/Vis spectroscopy was not possible. Nevertheless, we tried variable temperature (VT) NMR measurements as well as UV/Vis measurements knowing that whatever is observed is maybe not the cluster **1** itself but decomposition products or a mixture of both.^25^ Consequently, cluster **1** might be the first example of a model compound that demonstrates the primary chemical steps in photographic processing.

As previously reported,^26^ chemical stabilization of metalloid noble metal clusters by reaction with additional donor molecules or bulky organometallic substituents might be useful to stabilize the reactive cluster. However, all attempts in this direction have failed up so far, leading always to the formation of a silver mirror; investigations in this direction are still in progress in our lab.

NMR investigations of the crude reaction mixture, which was filtered from gray precipitate formed at room temperature after a reaction time of 12 hours, show one sharp resonance in the ^31^P NMR spectrum at −17 ppm (molecule **A**). Since the cluster is formed upon cooling this solution for at least ten days to −28 °C, we assigned this resonance first to cluster **1**. However, NMR measurements of isolated crystals dissolved in deuterated benzene show a fundamental different spectrum (Figure 4).

**Figure 4:**
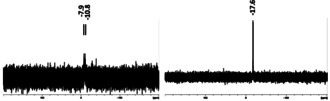
Room‐temperature ^31^P NMR spectra of **1** (left) and compound **A** (right).

The differences between **A** and **1** are also directly apparent upon inspection of the NMR tubes after the measurement. The solution of compound **1** darkened from red to dark brown with first signs of a silver mirror (Figure S6 in the Supporting Information). Beside this, the NMR tube of **A** remains orange‐brown and clear. These observations imply that the crude reaction mixture at room temperature does not contain **1** but another phosphorous‐containing substance. Several attempts to isolate **A** to get more information about this compound failed.^27^ Hence, up to now we have not been able to get further information about the specific role of **A** within the reaction system (side product or intermediate en route to **1**) and this aspect is still under investigation. Additionally, we conducted ^31^P VT‐NMR investigations on single crystals of **1** dissolved in [D_8_]toluene starting from low temperatures around −60 °C, resulting in broad resonances (Figure 5). The obtained signals change already on heating to ca. −20 °C, supporting the observation that a decomposition in solution already takes place at low temperatures. Considering all of this information about the sensitivity of **1**, it is fascinating that even one crystal of **1** is formed. During our synthetic improvements, we found that when the solution temperature does not exceed −20 °C, no gray precipitate is formed during the synthesis and no further workup procedure is necessary. Hence, during the synthesis under these conditions crystals of **1** can be obtained within 2 days with a yield of 68 % (with respect to Ag)! Due to the many modes of decomposition and with respect to the cluster size of 64 silver atoms, this is an extraordinary value.

**Figure 5:**
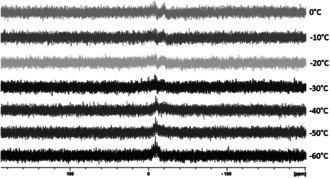
^31^P VT‐NMR of **1**. Temperature range: −60 °C to 0 °C.

To summarize, we were able to synthesize a rare example of a metalloid Ag cluster, namely Ag_64_(P^n^Bu_3_)_16_Cl_6_ (**1**), which is to our knowledge the first phosphine‐stabilized silver cluster without further protection from other organic or Group 16 based ligands. Within **1**, not a spherical but a brick‐shaped arrangement of the Ag atoms is observed, whereby structural relations to cubic (fcc) and hexagonal (hcp) close packing is observed. Additionally, **1** can be considered to be a 58 electron closed shell system, showing low thermal and photochemical stability. Due to the easy synthetic procedure starting from ^n^Bu_3_PAgCl, the high yield, and the good solubility in organic solvents, this cluster is a fruitful starting point for silver deposition, especially regarding its thermal and light sensitivity, which exceeds even that of silver halides.

## Conflict of interest

The authors declare no conflict of interest.

## Supporting information

As a service to our authors and readers, this journal provides supporting information supplied by the authors. Such materials are peer reviewed and may be re‐organized for online delivery, but are not copy‐edited or typeset. Technical support issues arising from supporting information (other than missing files) should be addressed to the authors.

Supplementary

## References

[1] A. F. Holleman , E. Wiberg , N. Wiberg Lehrbuch der Anorganischen Chemie, 103 ^rd^ ed., de Gruyter, Berlin, pp. 1722–1725.

[2] A. Schnepf , H. Schnöckel , Angew. Chem. Int. Ed. 2002, 41, 3532–3554; 10.1002/1521-3773(20021004)41:19<3532::AID-ANIE3532>3.0.CO;2-4 12370894

[3] The term “metalloid cluster” was introduced in 1999 by Schnöckel and co-workers to differentiate the different kinds of metal clusters fitting Cotton's widespread definition of “metal clusters” from 1964. Thereby a metalloid cluster exhibits more metal–metal than metal–ligand contacts, as well as so-called “naked” metal atoms showing metal–metal contacts only. This definition directly highlights the correlation of these clusters to the bulk phase of the corresponding metal. Although this term was mainly used for metalloid clusters of the main group metals, it already included all metals like Au, Ag, Pt etc. The term “metal cluster” or the later introduced term “metal nanocluster” do not include this depth of information. For further reading, see, for example, refs. [2, 4] and A. Purath , R. Köppe , H. Schnöckel , Angew. Chem. Int. Ed. 1999, 38, 2926–2928; 10.1002/(sici)1521-3773(19991004)38:19<2926::aid-anie2926>3.0.co;2-b 10540395

[4] A. Schnepf , Clusters – Contemporary Insight in Structure and Bonding, Vol. 174, Springer, Berlin, 2017, pp. 135–200.

[5] A. F. Holleman , E. Wiberg , N. Wiberg , Lehrbuch der Anorganischen Chemie, 103 ^rd^ ed., de Gruyter, Berlin, p. 1690.

[6] A. Schnepf , H. Schnöckel , Angew. Chem. Int. Ed. 2001, 40, 711–715; 11241599

[7] A. Schnepf , G. Stößer , R. Köppe , H. Schnöckel , Angew. Chem. Int. Ed. 2000, 39, 1637–1639; 10.1002/(sici)1521-3773(20000502)39:9<1637::aid-anie1637>3.0.co;2-o 10820460

[8] C. Schrenk , I. Schellenberg , R. Pöttgen , A. Schnepf , Dalton Trans. 2010, 39, 1872–1876. 20449434 10.1039/b921392a

[9] R. Jin , C. Zeng , M. Zhou , Y. Chen , Chem. Rev. 2016, 116, 10346–10413. 27585252 10.1021/acs.chemrev.5b00703

[10] J. Yang , R. Jin , ACS Mater. Lett. 2019, 1, 482–489.

[11] A. Desireddy , B. E. Conn , J. Guo , B. Yoon , R. N. Barnett , B. M. Monahan , K. Kirschbaum , W. P. Griffith , R. L. Whetten , U. Landman , T. P. Bigioni , Nature 2013, 501, 399–402. 24005327 10.1038/nature12523

[12] H. Yang , Y. Wang , X. Chen , X. Zhao , L. Gu , H. Huang , J. Yan , C. Xu , G. Li , J. Wu , A. Edwards , B. Dittrich , Z. Tang , D. Wang , L. Lehtovaara , H. Häkkinen , N. Zheng , Nat. Commun. 2016, 7:12809. 27611564 10.1038/ncomms12809 PMC5023969

[13] J. Yan , J. Zhang , X. Chen , S. Malola , B. Zhou , E. Selenius , X. Zhang , P. Yuan , G. Deng , K. Liu , H. Su , B. K. Teo , H. Häkkinen , L. Zheng , N. Zheng , Natl. Sci. Rev. 2018, 5, 694–702.

[14] F. Hu , J.-J. Li , Z.-J. Guan , S.-F. Yuan , Q.-M. Wang , Angew. Chem. Int. Ed. 2020, 59, 5312–5315; 10.1002/anie.201915168 31925894

[15] H. Schmidbaur , J. Adlkofer , K. Schwirten , Chem. Ber. 1972, 105, 3382–3388.

[16] M. R. Churchill , J. Donahue , F. J. Rotella , Inorg. Chem. 1976, 15, 2752–2758.

[17] Shortly after starting preparation under a light microscope, a darkening of the crystals from deep red to brown/black is observed. After several minutes, a silver mirror formed on the object plate where the crystals were located (Figure S8; Supporting Information).

[18] For details see the crystallographic section in the Supporting Information.

[19] C. Zeng , C. Liu , Y. Chen , N. L. Rosi , R. Jin , J. Am. Chem. Soc. 2016, 138, 8710–8713. 27355843 10.1021/jacs.6b04835

[20] M. J. Alhilaly , M. S. Bootharaju , C. P. Joshi , T. M. Besong , A.-H. Emwas , R. Juarez-Mosqueda , S. Kaappa , S. Malola , K. Adil , A. Shkurenko , H. Häkkinen , M. Eddaoudi , O. M. Baqr , J. Am. Chem. Soc. 2016, 138, 14727–14732. 27733038 10.1021/jacs.6b09007

[21] J. L. Persson , R. L. Whetten , H.-P. Cheng , R. S. Berry , Chem. Phys. Lett. 1991, 186, 215–222.

[22] Similar calculations were conducted for the metalloid silver cluster [Ag_67_(SR)_32_(PR_3_)_8_]^3+^: R. Juarez-Mosqueda , S. Kaappa , S. Malola , H. Häkkinen , J. Phys. Chem. C 2017, 121, 10698–10705.

[23] A complete overview of the highest lying orbitals of the superatom cluster down to HOMO−18 can be found in the Supporting Information.

[24] Due to the sensitivity of **1** further analysis beside single-crystal analysis is not possible to show the purity of the compound. However, a visual hint that only pure **1** is obtained can be drawn from the attempts at single-crystal X-ray diffraction. Thereby, plenty of crystals from different batches were examined to find one suitable for structure determination. The samples checked under the microscope that had been obtained from the reaction solution at temperatures below −20 °C consist of similar looking red crystalline rods only. Hence impurities, like Ag powder or mirror are not present. During these investigations all mounted crystals on the diffractometer show the same unit cell as that reported for **1**. A picture of the isolated single crystals is shown in Figure S5.

[25] UV/Vis spectra and some explanations can be found in the Supporting Information.

[26] M. Zhu , M. Li , C. Yao , N. Xia , Y. Yao , N. Yan , L. Liao , Z. Wu , Acta Phys. Chim. Sin. 2018, 34, 792–798;

[27] A detailed experimental section with two preparation pathways of **1** and **A** is given in the Supporting Information.

[28] Quantum-chemical calculations were carried out with the RI-DFT version of the Turbomole program package by employing the BP86-functional. The basis sets were of SVP quality. The TmoleX client was used as graphical user interface and for illustrating the molecular orbitals. Turbomole: O. Treutler , R. Ahlrichs , J. Chem. Phys. 1995, 102, 346—354; BP86 functional:

