# Supplementary material for: Synthesis and Characterization of the Highly Unstable Metalloid Cluster Ag64(PnBu3)16Cl6
Source: Angew Chem Int Ed Engl. 2020 Jul 1;59(34):14418–22. doi: 10.1002/anie.202006454 (PMC7496867; doi:10.1002/anie.202006454)
Supplement: Supplementary file 1 — Supplementary [file ANIE-59-14418-s001.pdf]

Angewandte Chemie
Eine Zeitschrift der Gesellschaft Deutscher Chemiker

Supporting Information

Synthesis and Characterization of the Highly Unstable Metalloid Cluster $Ag_{64}(P^nBu_3)_{16}Cl_6$

*Maximilian Diecke, Claudio Schrenk, and Andreas Schnepf**

anie_202006454_sm_miscellaneous_information.pdf

Content

1. Experimental 2
2. Crystal data and structure determination of **1** 4
3. UV/Vis measuements 8
4. Instability of **1** 9
5. Quantum chemical calculations 11
6. References 18

1. Experimental

All experiments were performed under a nitrogen atmosphere by using standard Schlenk techniques. All solvents were pre-dried over sodium/benzophenone and distilled prior to use. Deuterated solvents were dried oven 3Å molecular sieves. $^{n}Bu_3PAgCl$ was prepared after a modified literature procedure[1] and was stored under light exclusion as a 1 M solution in toluene.

Synthesis of $Ag_{64}(P^{n}Bu_3)_{16}Cl_6$ (1):

5 mL of 1 M toluene solution of $^{n}Bu_3PAgCl$ (5 mmol, 1eq) was diluted with toluene to a volume of 100 mL under rigorous light exclusion and cooled to −78°C. 3.5 ml (3.5 mmol, 0.7 eq) of a 1 M solution of LiBH(*sec*-Bu)$_3$ in THF was added resulting in a pale yellow reaction color. The mixture was stirred 5 h by slowly warming up to −20°C. The now dark red solution was stored at −28°C for 2 days, to give dark red single crystals of **1** (560 mg, 67% yield with respect to Ag). A second, similar synthetic route is given in the supporting information. ^{1}H-NMR (D8-Toluene): 0.95 (m, br, 9H, CH_3) 1.25 (m, br., 6H, P-CH_2) 1.50 (m, br., 12H, C_2H_4-CH_3), ^{31}P{^{1}H}-NMR (D8-Toluene) −9.0 (br., $P^{n}Bu_3$)

Alternative synthetic route

5 mL of 1 M toluene solution of $^{n}Bu_3PAgCl$ (5 mmol, 1eq) was diluted with toluene to a volume of 100 mL under rigorous light exclusion and cooled to -78°C. 3.5 ml (3.5 mmol, 0.7 eq) of a 1 M solution of LiBH(*sec*-Bu)$_3$ in THF was added resulting in a pale yellow reaction color. The mixture was stirred 12 h by slowly warming up to room temperature. A lot of gray precipitate was formed during warm-up. The mixture was filtered. NMR investigations showed one single resonance in the ^{31}P-NMR which was assigned to the unknown species **A**.

Compound **A**: ^{31}P{^{1}H}-NMR (C_6D_6) -17.7 (s, $P^{n}Bu_3$)

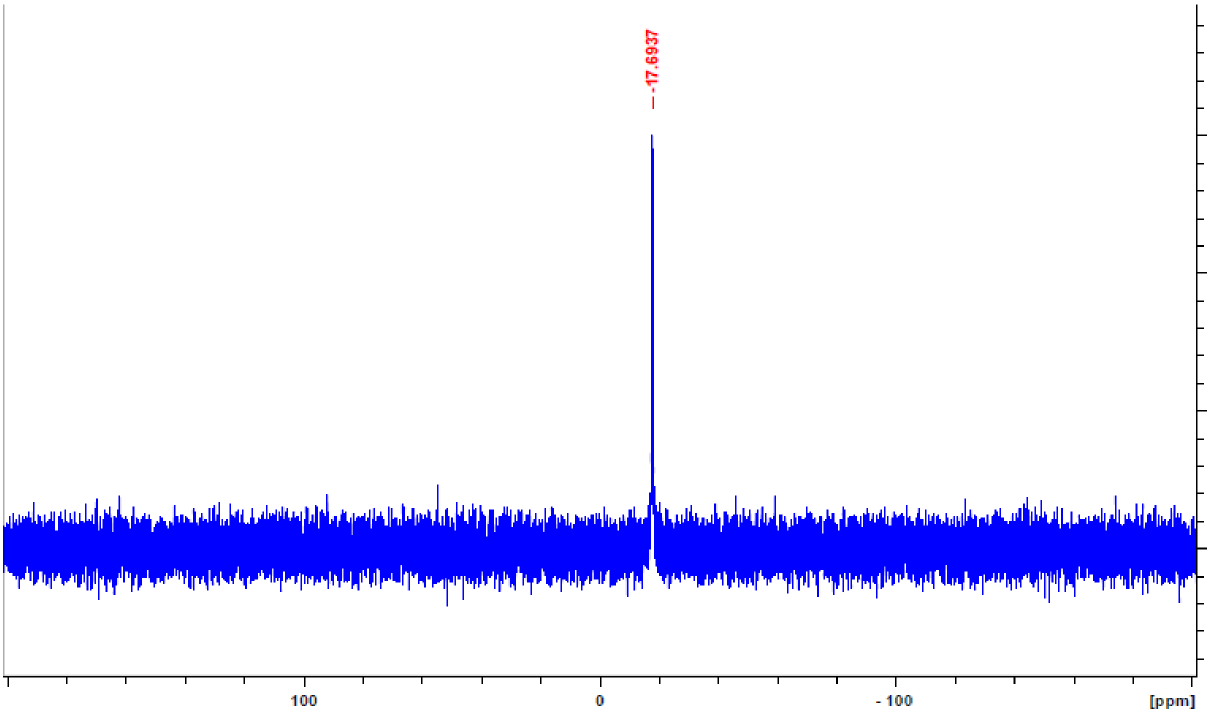

Figure S1: ^{31}P-NMR of **A**

The dark brown toluene solution containing **A** was stored at -28°C for 2 weeks, to give dark red single crystals of **1** (30 mg, 4% yield with respect to Ag). ^{1}H-NMR (D8-Toluene): 0.95 (m, br, 9H, C*H_3*) 1.25 (m, br., 6H, P-C*H_2*) 1.50 (m, br., 12H, C_2*H_4*-CH_3), ^{31}P{^{1}H}-NMR (D8-Toluene) -9.0 (br., *P*^{n}Bu_3)

2. Crystal data and structure determination of **1**

2.1 Crystallography

Crystals were mounted on the diffractometer at 100 K. The data were collected on a Bruker APEX II DUO diffractometer equipped with an IμS microfocus sealed tube and QUAZAR optics for monochromated MoKα radiation (λ = 0.71073 Å) and equipped with an Oxford Cryosystems cryostat. A semiempirical absorption correction was applied using the program SADABS. The structure was solved by direct methods and refined against F^2 for all observed reflections. Programs used: SHELXS and SHELXL[2] within the Olex2 program package.[3] Due to the high instability, crystals of **1** were prepared in a dark room under a light microscope with only a few seconds illumination time. Preparing under light lead to formation of a silver mirror on the object plate. Measurements at 150 K and 200 K lead to loss of crystallinity due to loss of crystal solvent. Most of the crystals could be visually identified as twins. The used crystal was also twinned, but not visually resolvable. Therefore, the crystal was refined as physical twin with two domains with a batch scaling factor of 0.3856. During the complete measurement time, the room was darkened to prevent from degradation.

Crystal structure data for **1**: $C_{192}H_{432}Ag_{64}Cl_6P_{16}$, M = 10353.25 g mol^{-1}, T = 100 K, triclinic, $P\bar{1}$, a = 20.1009(17) Å, b = 21.7488(19) Å, c = 24.963(2) Å, α = 80.8070(18)°, β = 67.2830(18)°, γ = 72.2570(18)°, V = 9576.6(14) Å^3, Z = 1, ρ_{calc} = 1.795 g cm^{-3}, μ = 3.318 mm^{-1}, F(000) = 4934.0, crystal dimensions: 0.311 mm × 0.079 mm × 0.072 mm; $2\Theta_{max}$ = 52.96°, 56790 independent reflections (R_{int} = 0.0897), $R_1(I>2\sigma(I))$ = 0.0668, wR_2(all refl.) = 0.2011. The H atom positions in all compounds were refined using a riding model. To refine the co crystallized solvent molecules, SQUEEZE[4] was used to identify a volume of 2625 Å^3, where 1051 electrons were found. This fits almost perfectly to 21 toluene molecules in the whole unit cell.

CCDC-2000939 contains the supplementary crystallographic data of **1** and can be obtained online free of charge at www.ccdc.cam.ac.uk/conts/retrieving.html or from Cambridge Crystallographic Data Centre, 12 Union Road, Cambridge CB21EZ; Fax: (+44)1223-336-033; or deposit@ccdc.cam.ac.uk.

2.2 Bond lengths and angles

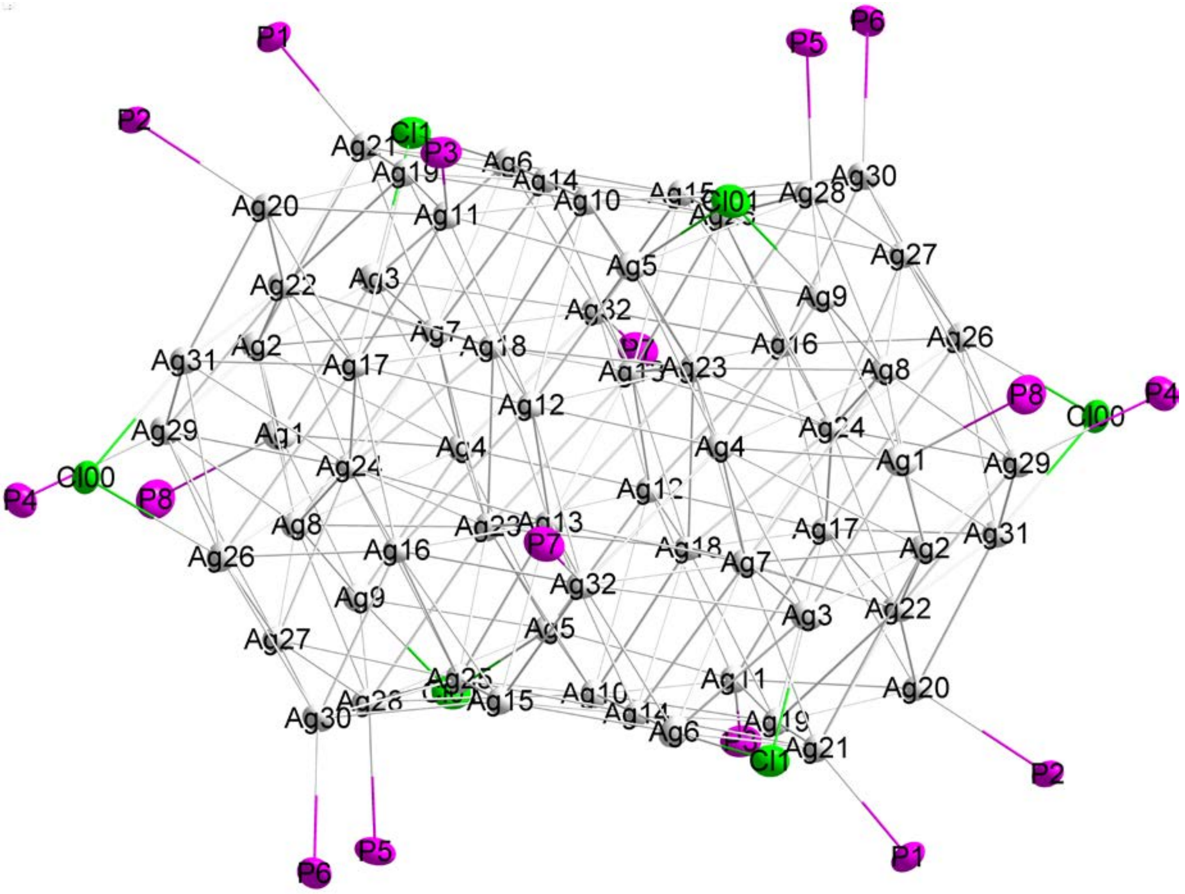

Figure S2: Atom names of cluster **1** excluding C and H

Bond lengths [Å]

Ag1	Ag2	2.7986(17)
Ag1	Ag3	2.8765(18)
Ag2	Ag3	2.8005(16)
Ag2	Ag7	2.9189(16)
Ag3	Ag4	2.9889(15)
Ag3	Ag6	2.9975(18)
Ag4	Ag5	3.1321(15)
Ag4	Ag7	2.8799(16)
Ag5	Ag9	3.0090(17)
Ag5	Ag10	2.7960(16)
Ag6	Ag7	3.2290(16)
Ag6	Ag14	2.8789(16)
Ag6	Cl1	2.645(4)
Ag7	Ag13	3.2166(16)
Ag7	Ag14	3.1812(16)
Ag8	Ag9	2.8605(16)
Ag8	Ag23[1]	3.0866(16)
Ag9	Ag28[1]	2.9185(17)
Ag9	Cl01	2.557(4)
Ag10	Ag11	2.9607(15)
Ag10	Ag14[1]	3.0266(16)
Ag11	Ag12	3.2541(15)
Ag11	Ag17	2.9657(16)
Ag12	Ag13	2.9084(15)
Ag12	Ag16	3.1270(16)
Ag13	Ag14	3.2986(14)
Ag13	Ag15	3.2629(15)
Ag14	Ag10[1]	3.0266(16)
Ag14	Ag15	2.9428(15)
Ag15	Ag16	2.8417(15)
Ag15	Ag25	2.9399(16)
Ag16	Ag17	2.9445(14)
Ag16	Ag24	3.0963(16)
Ag17	Ag18	3.1261(15)
Ag17	Ag20	2.8448(14)
Ag18	Ag7[1]	2.9581(16)
Ag18	Ag13[1]	2.9678(15)
Ag19	Ag14[1]	3.0491(15)
Ag19	Ag20	2.8171(15)
Ag20	Ag31	2.9409(16)
Ag20	P2	2.439(4)
Ag21	Ag6[1]	3.0449(15)
Ag21	Ag7[1]	3.1374(15)
Ag22	Ag2[1]	2.9606(16)

Ag22	Ag7[1]	3.1249(15)
Ag23	Ag4[1]	3.0860(16)
Ag23	Ag7[1]	3.1861(15)
Ag24	Ag8[1]	3.0252(16)
Ag24	Ag26	3.0594(15)
Ag25	Ag10[1]	2.9990(15)
Ag25	Ag27	2.8950(16)
Ag26	Ag27	2.7689(16)
Ag26	Ag30	2.9256(16)
Ag27	Ag8[1]	2.9446(16)
Ag27	Ag28	2.8971(16)
Ag27	Ag30	2.8158(17)
Ag28	Ag8[1]	2.9708(15)
Ag28	Ag9[1]	2.9185(17)
Ag28	P5	2.471(4)
Ag29	Ag2[1]	2.8803(15)
Ag29	Ag8[1]	2.9298(15)
Ag30	P6	2.456(4)
Ag31	Cl00	2.581(4)
Ag32	P7	2.441(4)

Bond angles [°]

Ag2	Ag1	Ag3	59.12(4)
Ag2	Ag1	Ag4	81.47(5)
Ag1	Ag2	Ag3	61.83(4)
Ag1	Ag2	Ag7	99.68(5)
Ag1	Ag3	Ag4	61.98(4)
Ag1	Ag3	Ag6	159.30(5)
Ag1	Ag4	Ag5	116.97(5)
Ag1	Ag4	Ag12	170.07(5)
Ag9	Ag5	Ag4	55.39(4)
Ag9	Ag5	Ag11	172.63(5)
Ag3	Ag6	Ag7	57.19(4)
Ag3	Ag6	Ag21[1]	79.66(4)
Ag2	Ag7	Ag3	56.60(4)
Ag2	Ag7	Ag6	104.63(4)
Ag2	Ag8	Ag1	52.46(3)
Ag2	Ag8	Ag23[1]	105.85(5)
Ag1	Ag9	Ag5	125.50(5)
Ag1	Ag9	Ag28[1]	132.89(5)
Ag5	Ag10	Ag11	63.19(4)
Ag5	Ag10	Ag14[1]	164.45(5)
Ag5	Ag11	Ag12	53.40(3)
Ag5	Ag11	Ag20	163.48(5)
Ag4	Ag12	Ag11	122.50(5)
Ag4	Ag12	Ag16	121.88(4)
Ag7	Ag13	Ag14	58.44(3)
Ag7	Ag13	Ag15	86.67(4)
Ag6	Ag14	Ag7	64.17(4)
Ag6	Ag14	Ag10[1]	179.23(5)
Ag6	Ag15	Ag13	92.65(4)
Ag6	Ag15	Ag14	59.54(4)
Ag32	Ag15	Ag30	128.17(5)
Ag13	Ag16	Ag12	57.17(4)
Ag13	Ag16	Ag17	90.01(4)
Ag11	Ag17	Ag18	83.26(4)
Ag11	Ag17	Ag24	139.54(5)
Ag7[1]	Ag18	Ag10	122.22(5)
Ag7[1]	Ag18	Ag12	166.25(5)
Ag10	Ag19	Ag22	130.81(5)
Ag11	Ag19	Ag10	59.94(4)
Ag17	Ag20	Ag11	60.44(4)
Ag17	Ag20	Ag31	59.72(4)
Ag61	Ag21	Ag71	62.95(4)
Ag141	Ag21	Ag61	57.38(4)
Ag21	Ag22	Ag71	57.25(4)
Ag21	Ag22	Ag19	138.60(5)
Ag41	Ag23	Ag71	54.64(3)
Ag101	Ag23	Ag131	104.91(4)
Ag81	Ag24	Ag16	137.88(5)
Ag81	Ag24	Ag22	89.91(4)
Ag101	Ag25	Ag13	92.26(4)
Ag101	Ag25	Ag30	164.64(5)
Ag16	Ag26	Ag24	62.90(4)
Ag16	Ag26	Ag30	61.60(4)
Ag24	Ag27	Ag81	61.83(4)
Ag25	Ag27	Ag81	94.40(4)
Ag91	Ag28	Ag81	58.11(4)
Ag25	Ag28	Ag81	93.99(4)
Ag21	Ag29	Ag81	60.11(4)
Ag21	Ag29	Ag22	60.55(4)
Ag15	Ag30	Ag16	57.92(4)
Ag15	Ag30	Ag25	57.08(4)
Ag17	Ag31	Ag20	58.48(4)
Ag17	Ag31	Ag24	56.49(3)
Ag4	Ag32	Ag12	60.50(4)
Ag6	Ag32	Ag4	99.54(5)

2.3 Comparison to fcc and hcp

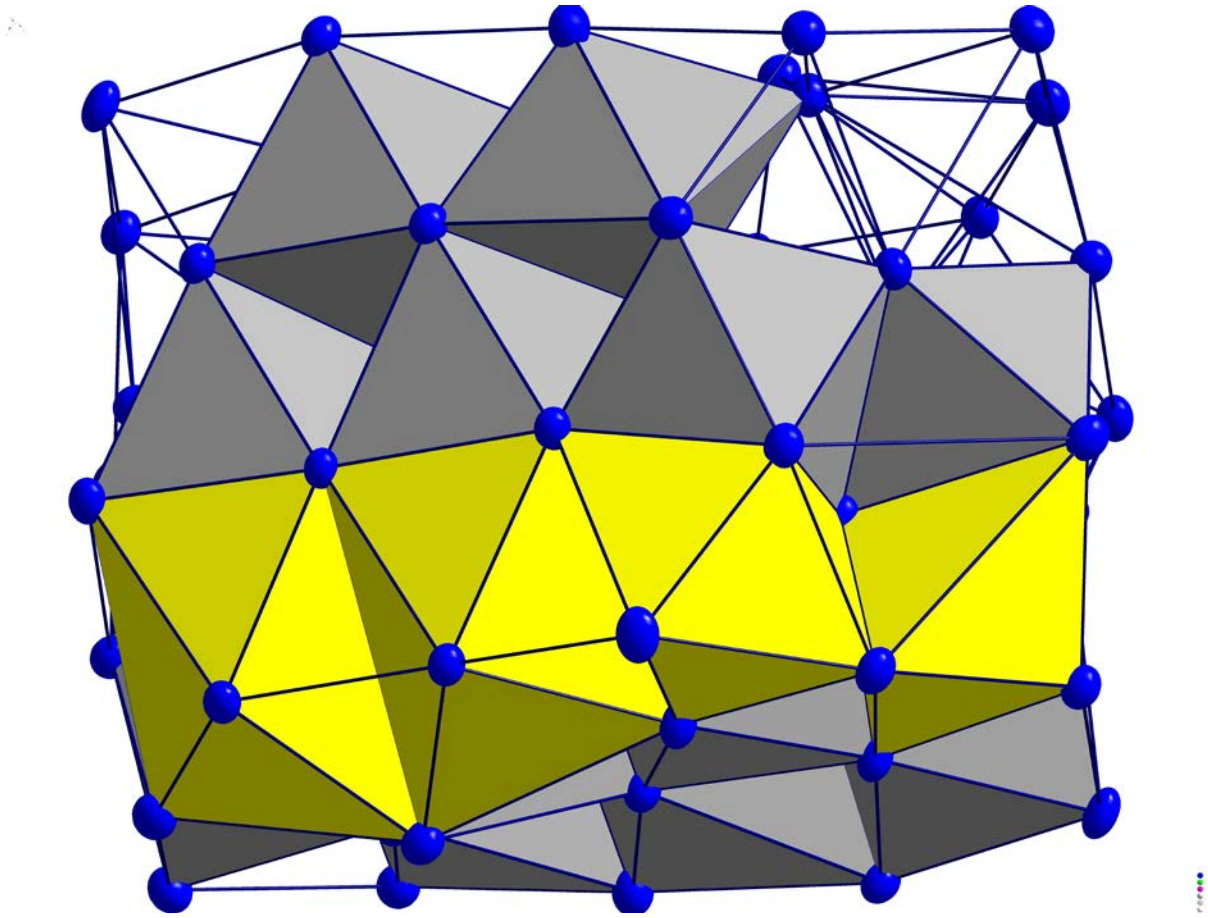

Figure S3: yellow: Ag_6 octahedra face-sharing to other Au6 octahedra (hcp like), gray: Ag_6 octahedra edge-sharing to other gray octahedra (fcc like) and face-sharing to yellow octahedra

3. UV/Vis measuements

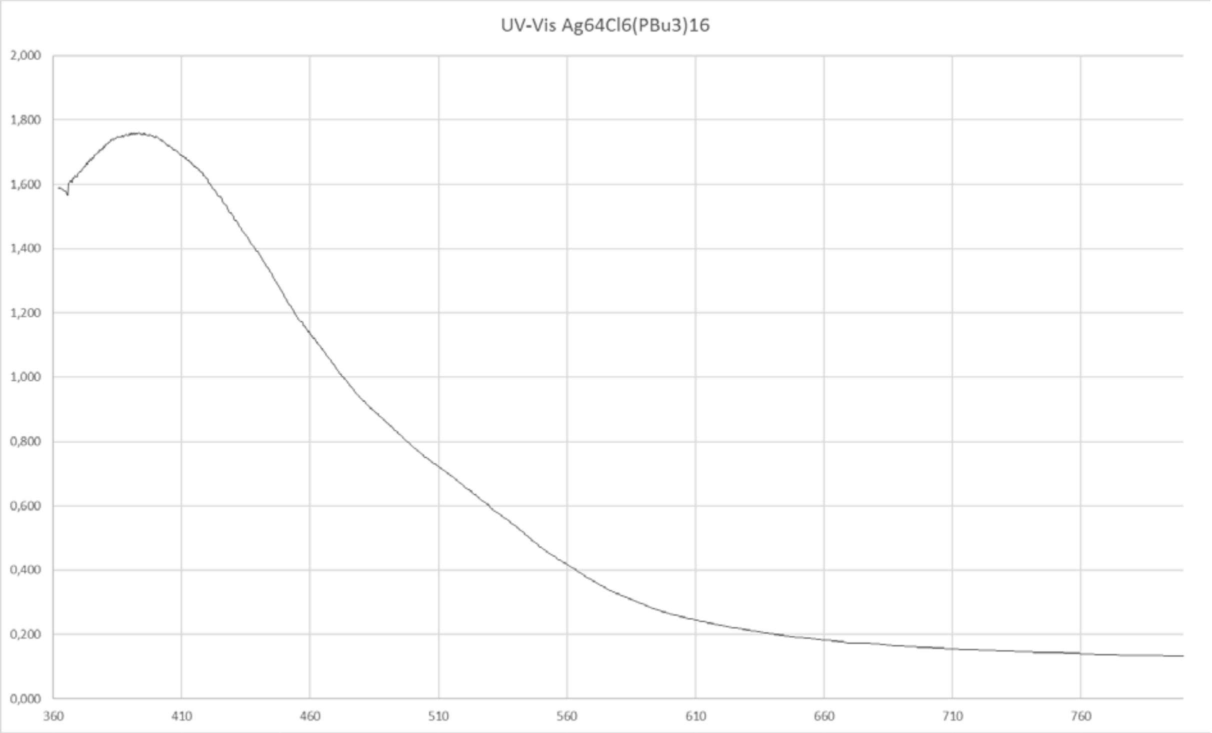

Figure S4: UV/Vis spectra of a toluene solution of **1** at room temperature. Due to the instability of **1**, no further results could be discussed, since a degradation (darkening of the solution) is observed during the measurement process.

UV/Vis results [nm]:

394.0 (br); 417.6 (shoulder); 442.4 (shoulder); 466.0 (shoulder); 525.6 (shoulder); 564.8 (shoulder),

4. Instability of **1**

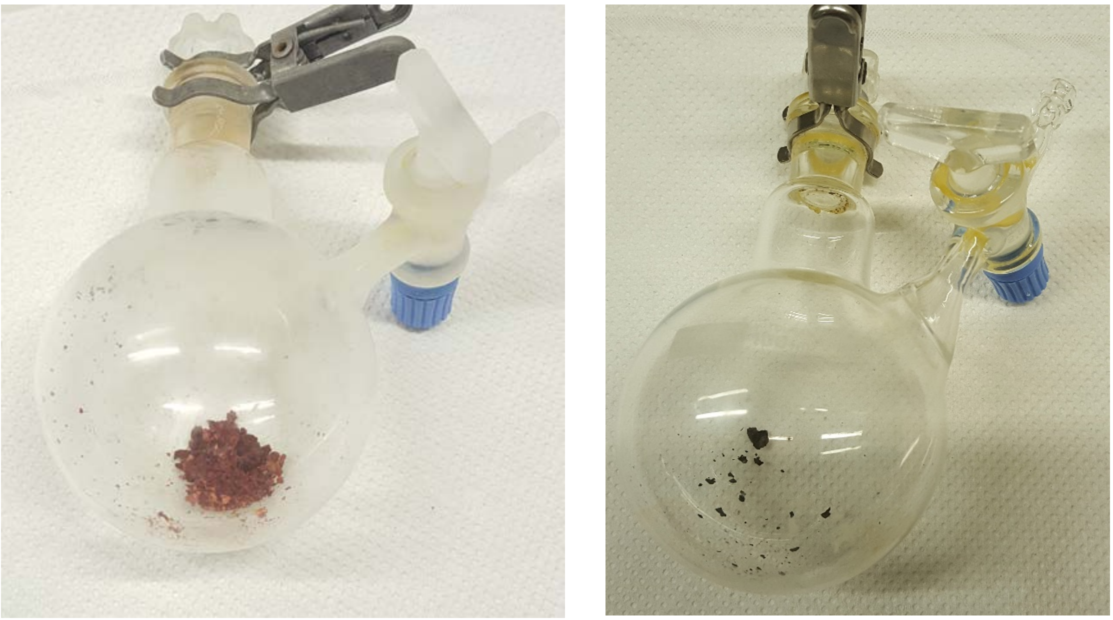

Figure S5: Crystals of **1** at -28°C (left) and at r.t. (right) after storage for 7 days under a nitrogen atmosphere.

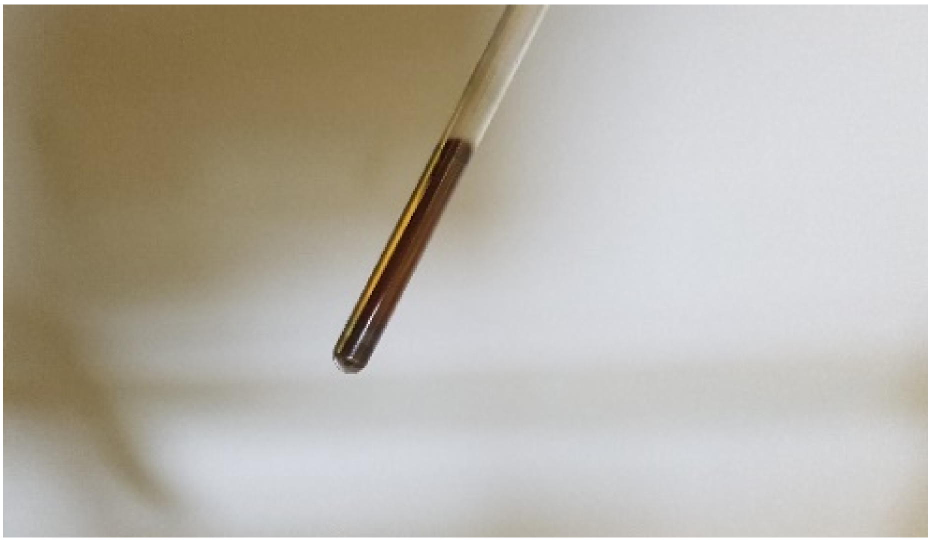

Figure S6: NMR tube of a solution of **1** directly after the measurement at r.t.

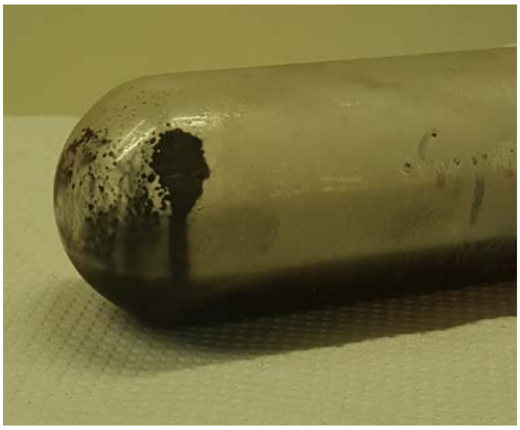

Figure S7: Crystals of **1** and Ag impurity during the synthesis via compound **A**

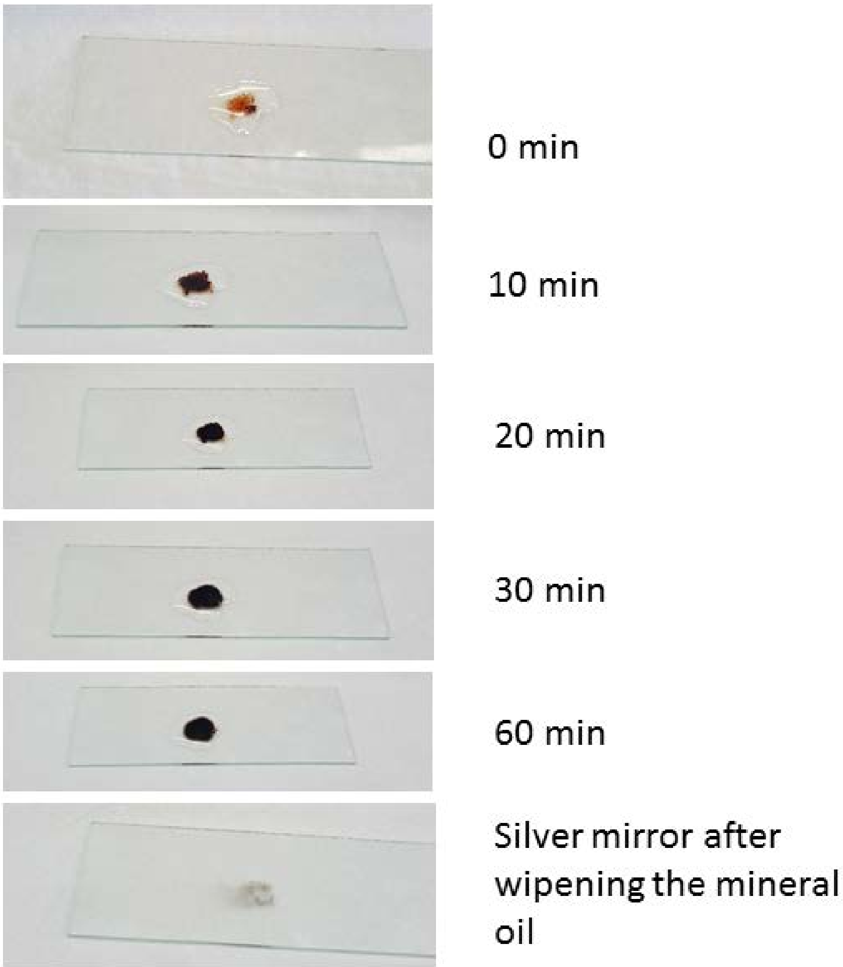

Figure S8: Object slide with crystals of **1** in mineral oil directly after transferring from a −30°C cold schlenk vessel (0 min); illustrated in steps of 10 minutes. Last picture shows the silver mirror on the object slide after the mineral oil is wiped off.

5. Quantum chemical calculations

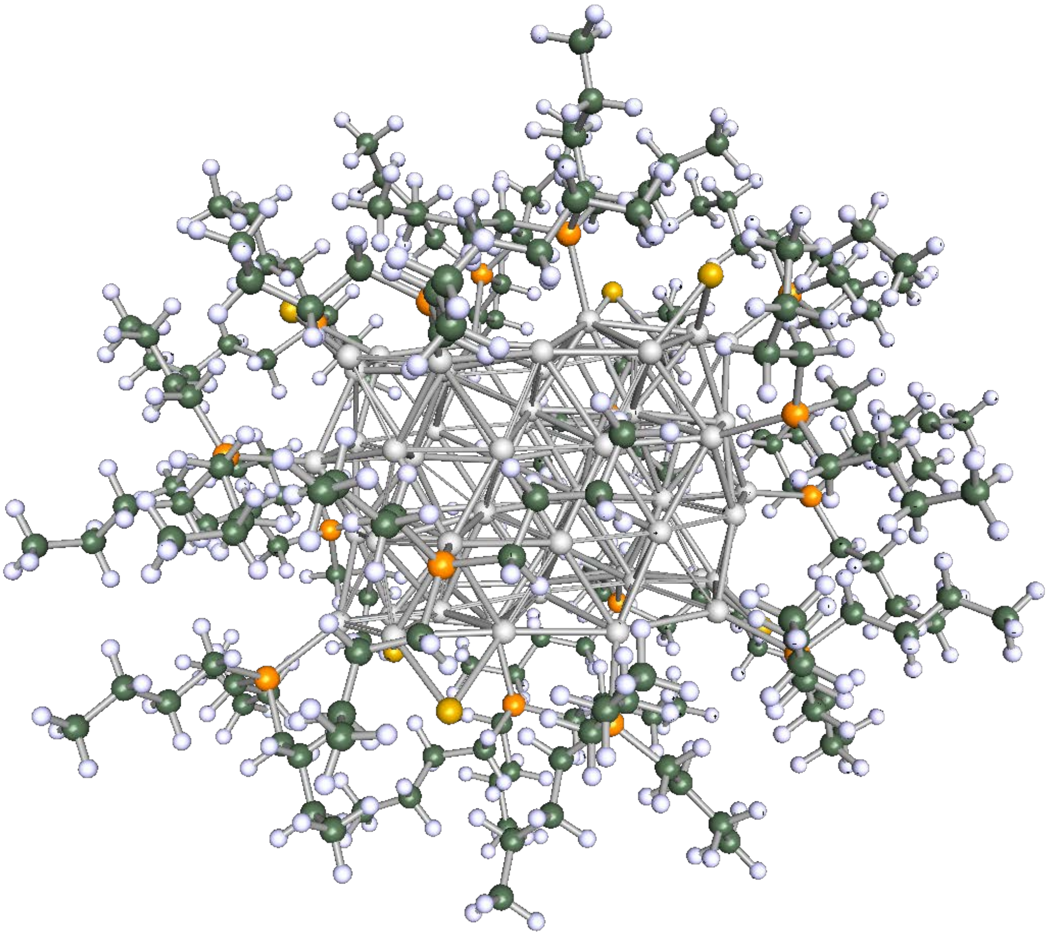

Figure S9: Geometry optimized structure of **1**[5]

Point group:	C_1
Energy:	-25208.79775684243 Hartree
HOMO-LUMO-gap:	0.066 eV

Atomic coordinates:

```
Ag -2.453917 -0.012963 -6.666056
Ag -2.386042 2.039853 -4.577601
Ag 0.031293 1.362021 -5.950936
Ag -0.715802 -1.060068 -4.690616
Ag -3.538347 -0.483129 -4.103267
Ag -2.818607 -2.646573 -5.685462
P -3.109989 0.084633 -9.068123
Ag -0.086652 1.198911 -3.081798
Ag -0.347201 4.022329 -3.758778
Ag -3.009380 4.024506 -2.620144
Ag -5.168565 1.870037 -3.553588
Ag 2.156169 2.711112 -4.451586
Ag 2.082367 -0.214460 -4.294259
Cl 2.072355 2.581711 -7.091775
Ag -1.004863 -3.888283 -3.792241
Ag 0.782177 -2.145350 -2.532102
Ag -1.685335 -1.001499 -1.714460
Ag -3.032475 1.393842 -1.781163
Ag -5.137627 -0.631924 -1.817326
Ag -3.624708 -3.005380 -2.789879
Cl -1.824380 -5.029759 -6.120451
C -2.364922 -1.293096 -10.091836
C -2.688604 1.598278 -10.086034
C -4.951902 -0.109436 -9.350040
Ag 1.983192 0.120390 -1.321304
Ag 1.628188 3.066641 -1.765193
Ag -0.390011 1.413942 -0.330286
Ag -0.782474 4.323258 -0.882801
P -0.339549 5.897781 -5.488655
Ag -2.746596 2.912598 0.646226
Ag -3.255614 5.548788 -0.184120
Ag -5.112087 3.225485 -0.890291
Ag -7.003060 1.022499 -0.654640
P -7.358781 2.099806 -4.811111
Ag 4.058200 1.496034 -2.621186
Ag 4.537402 -0.719033 -0.695154
P 3.783193 -1.249721 -5.818173
Ag -1.249862 -3.682834 -1.005765
Ag 1.229481 -5.005206 -1.945517
Ag 2.746591 -2.912634 -0.646368
Ag 0.390068 -1.413971 0.330194
Ag -1.983091 -0.120397 1.321321
Ag -3.631440 -2.232197 -0.043092
Ag -4.537319 0.718794 0.695101
Ag -6.354583 -1.370046 0.731838
P -5.115547 -5.041084 -3.124349
C -2.807187 -2.713600 -9.701894
C -1.214684 2.028754 -9.975378
C -5.455653 -0.258299 -10.795396
Ag 1.685384 1.001369 1.714251
Ag 3.032509 -1.393966 1.781059
```

Ag 3.631396 2.232257 0.043058
Ag 1.249867 3.682729 1.005699
Ag -0.782138 2.145337 2.531979
Ag -1.229483 5.005149 1.945438
C -0.646073 7.625234 -4.824033
C 1.125973 6.127921 -6.623267
C -1.690377 5.674518 -6.768216
P -4.371632 7.749819 -0.640153
Cl -7.760008 3.505155 -0.670221
C -8.707913 3.213338 -4.156722
C -8.278959 0.473219 -4.876495
C -7.204404 2.595994 -6.611533
Ag 6.354512 1.369957 -0.731834
Ag 7.003101 -1.022642 0.654612
C 4.846093 -2.555109 -4.995110
C 5.003681 -0.162203 -6.725424
C 2.976788 -2.223717 -7.197957
Ag -1.628172 -3.066595 1.765208
Ag 0.782545 -4.323241 0.882801
Ag 3.255589 -5.548855 0.184101
P 1.422662 -6.697777 -3.858415
Ag 3.009430 -4.024658 2.620228
Ag 5.112099 -3.225649 0.890261
Ag 0.086811 -1.198889 3.081748
Ag -4.058280 -1.495996 2.621181
Ag -2.082390 0.214490 4.294198
P -8.406802 -2.431029 1.679417
C -5.053859 -6.010600 -4.720615
C -4.704236 -6.373101 -1.877756
C -6.940562 -4.708794 -2.876912
C -2.138332 -3.797224 -10.567429
C -0.804491 3.139848 -10.962382
C -6.975797 -0.489080 -10.883960
Ag 0.715825 1.060082 4.690575
Ag 1.004809 3.888258 3.792180
Ag 3.538366 0.483046 4.103175
Ag 3.624635 3.005192 2.789879
Ag 5.137573 0.631818 1.817174
Ag 5.168611 -1.870193 3.553564
P -1.422446 6.697702 3.858343
C -0.663131 8.781075 -5.838511
C 2.454206 6.453397 -5.919442
C -3.117170 5.980447 -6.293310
C -3.317835 9.236120 -0.216534
C -4.791463 8.004564 -2.445771
C -5.954777 8.179960 0.260581
C -8.450322 4.719059 -4.308480
C -7.527906 -0.667886 -5.579893
C -8.479816 2.596893 -7.470637
P 8.406809 2.430860 -1.679548
Cl 7.760018 -3.505361 0.669980
C 6.030309 -1.993400 -4.193108
C 5.948169 -0.853462 -7.723586
C 2.398880 -1.368267 -8.335730
Ag -2.156198 -2.711059 4.451591
Ag 0.347155 -4.022407 3.758871
P 4.371645 -7.749956 0.640137
C -0.117743 -7.712032 -4.158415
C 1.705549 -5.896807 -5.526383
C 2.742186 -8.037000 -3.822494
Ag 2.386096 -2.039925 4.577636
Ag -0.031313 -1.361984 5.950886
P -3.783190 1.249837 5.818012
C -8.792340 -4.203793 1.212502
C -8.509067 -2.431142 3.543982
C -9.957507 -1.479790 1.246683
C -5.646296 -5.277785 -5.934259
C -4.947488 -5.981637 -0.412386
C -7.906991 -5.900217 -2.979629
C -2.574893 -5.222325 -10.203463
C -1.572868 4.459942 -10.812816
C -7.482314 -0.653938 -12.322878
Ag 2.453946 0.012885 6.666031
Ag 2.818594 2.646521 5.685449
Cl 1.824091 5.029674 6.120413
P 5.115412 5.041028 3.124442
P 7.359029 -2.099842 4.811031
C 0.117875 7.712058 4.158411
C -1.705403 5.896794 5.526321
C -2.742037 8.036847 3.822367
C -1.111256 10.124154 -5.234782
C 3.515402 7.053668 -6.865581
C -4.176758 5.693693 -7.371921
C -1.938713 9.263214 -0.892786
C -5.360071 9.371237 -2.860983
C -7.185372 7.350755 -0.139357
C -9.596058 5.565012 -3.725342
C -8.342424 -1.972852 -5.678429
C -8.259850 3.172931 -8.882059
C 8.792416 4.203643 -1.212708
C 8.509126 2.430881 -3.544126
C 9.957517 1.479613 -1.246753
C 6.887394 -3.081690 -3.522730
C 6.888380 0.123127 -8.453851
C 1.830163 -2.212530 -9.490336
Cl -2.072555 -2.581337 7.091759
P 0.339606 -5.897787 5.488938
C 3.317742 -9.236199 0.216548
C 4.791454 -8.004674 2.445767
C 5.954787 -8.180179 -0.260588
C -0.688251 -8.429614 -2.926575
C 1.752282 -6.815448 -6.758158
C 4.181250 -7.554660 -4.064458
C -4.845976 2.555237 4.994838
C -5.003725 0.162311 6.725163
C -2.976897 2.223825 7.197848
C -10.202362 -1.320943 -0.262406
C -10.039614 -4.863673 1.822747
C -7.458524 -3.310519 4.237863
C -5.482713 -6.039796 -7.264334
C -4.399193 -7.014926 0.586422
C -9.389168 -5.485662 -2.928037
P 3.109708 -0.084474 9.068232
C 5.053617 6.010555 4.720715
C 4.704026 6.373034 1.877830
C 6.940470 4.708860 2.877049
C 8.708107 -3.213413 4.156520
C 8.279206 -0.473224 4.876336
C 7.204758 -2.596011 6.611481
C 0.688380 8.429786 2.926649
C -1.752319 6.815485 6.758057
C -4.181100 7.554435 4.064227
C -1.091501 11.281137 -6.242170
C 3.826800 6.214497 -8.111921
C -5.569653 6.216958 -7.002071
C -1.097269 10.482040 -0.473361
C -5.703977 9.455633 -4.359041
C -8.409722 7.655030 0.744038
C -9.409809 7.072643 -3.935287
C -9.546144 -1.915751 -6.630903
C -9.526331 3.164179 -9.748389
C 10.202393 1.320917 0.262345
C 10.039694 4.863464 -1.823002
C 7.458853 3.310535 -4.238067
C 8.205758 -2.532829 -2.964633
C 7.805861 -0.565957 -9.472162
C 1.547507 -1.387051 -10.752060
C 0.646123 -7.625266 4.824377
C -1.125904 -6.127838 6.623589
C 1.690496 -5.674430 6.768430
C 1.938693 -9.263227 0.892941
C 5.359926 -9.371379 2.861030
C 7.185407 -7.351039 0.139395
C -1.959448 -9.229670 -3.267099
C 2.044153 -6.053614 -8.063137
C 5.179886 -8.716115 -4.227398
C -6.030205 1.993530 4.192841
C -5.948259 0.853564 7.723289
C -2.398984 1.368384 8.335626
C -11.467363 -0.512602 -0.616957
C -10.227722 -6.325630 1.375945
C -7.603738 -3.311770 5.768220
C -6.243610 -7.371194 -7.346992
C -4.649251 -6.627819 2.049293
C -10.358377 -6.668403 -3.052901
C -11.466109 0.937093 -0.111076
C -11.467106 -6.993380 1.986316
C -6.525828 -4.136843 6.479291
C 5.646087 5.277768 5.934362
C 5.482582 6.039844 7.264413
C 6.243553 7.371207 7.346989
C 4.947762 5.981787 0.412480
C 4.399175 7.014925 -0.586338
C 4.649351 6.627879 -2.049215
C 7.906810 5.900347 2.979818
C 9.389013 5.485925 2.927858
C 10.358151 6.668733 3.052673
C 8.450469 -4.719115 4.308337
C 9.595933 -5.565146 3.724787
C 9.409454 -7.072786 3.934453
C 7.528255 0.667782 5.579991
C 8.342709 1.972796 5.678418
C 9.546687 1.915707 6.630567
C 8.480217 -2.596843 7.470501
C 8.260343 -3.172743 8.881991
C 9.526888 -3.163933 9.748227
C 11.467519 0.512800 0.616960
C 11.466378 -0.937014 0.111414
C 10.227864 6.325420 -1.376206
C 11.467241 6.993138 -1.986629
C 7.604014 3.311562 -5.768431
C 6.526231 4.136758 -6.479559
C 1.097258 -10.482163 0.473839
C -0.286208 -10.528201 1.133482
C 5.703859 -9.455731 4.359082
C 6.268250 -10.820147 4.776542
C 8.409818 -7.655471 -0.743855
C 9.674083 -6.897840 -0.319229
C -2.557869 -9.989779 -2.077631
C 2.084515 -6.963105 -9.297882
C 6.595198 -8.256287 -4.599687
C 0.663170 -8.781089 5.838862
C 1.111191 -10.124190 5.235116
C 1.091433 -11.281171 6.242502
C -2.454142 -6.453350 5.919817
C -3.515324 -7.053537 6.866018
C -3.826677 -6.214289 8.112310
C 3.117243 -5.980429 6.293456
C 4.176898 -5.693734 7.372007
C 5.569736 -6.217109 7.002108
C -6.887136 3.081782 3.522222
C -8.205526 2.532950 2.964149
C -6.888490 -0.123033 8.453511
C -7.806014 0.566041 9.471789
C -1.830464 2.212660 9.490322
C -1.547920 1.387178 10.752067
C 2.364467 1.293275 10.091802
C 2.688230 -1.598108 10.086140
C 4.951586 0.109633 9.350391
C 2.806931 2.713750 9.701983
C 1.214360 -2.028670 9.975208
C 5.455154 0.258497 10.795808
C 2.137979 3.797422 10.567383
C 0.804021 -3.139733 10.962179
C 6.975284 0.489302 10.884551
C 2.574717 5.222489 10.203489
C 1.572508 -4.459789 10.812858
C 7.481634 0.654186 12.323524
C 0.286361 10.528023 -1.132672
C -6.268481 10.820024 -4.776437
C -9.673931 6.897189 0.319612
C 1.959585 9.229782 3.267306
C 2.558133 9.989943 2.077930
C -2.044208 6.053675 8.063045
C -2.084742 6.963210 9.297753
C -5.179762 8.715855 4.227297
C -6.595043 8.255972 4.599643
H -2.060433 9.271646 -2.000895
H -1.390269 8.322321 -0.651485
H -6.277638 9.597216 -2.268947
H -4.629738 10.176903 -2.616364
H -3.857708 7.773542 -3.008864
H -5.496892 7.182542 -2.705537
H -6.507604 10.845667 -5.862485

H -5.543270 11.639895 -4.572127
H -7.204647 11.057010 -4.221888
H -9.995571 7.194842 -0.703837
H -9.495514 5.799236 0.306672
H -10.520467 7.105163 1.011358
H -0.099624 8.450295 4.963774
H 0.867159 6.996043 4.570019
H -2.666569 8.539699 2.831628
H -2.464320 8.798043 4.588070
H -5.211956 9.316119 3.287726
H -4.799850 9.408976 5.014962
H -7.287943 9.119374 4.708031
H -6.596342 7.699220 5.563708
H -7.021005 7.579061 3.825630
H -3.889406 10.165425 -0.445349
H -3.191662 9.208022 0.890246
H -8.161440 7.397255 1.800302
H -8.607451 8.754036 0.736826
H -9.349147 7.330956 -5.017234
H -8.472564 7.432370 -3.453326
H -10.253497 7.651816 -3.498265
H 2.839109 9.296931 1.253545
H 3.473558 10.549222 2.373900
H 1.835520 10.727422 1.661402
H 0.863751 11.420172 -0.804294
H 0.202885 10.571570 -2.241790
H 0.885447 9.625936 -0.877947
H -6.436770 8.654764 -4.610552
H -4.788420 9.229922 -4.955761
H -3.016315 5.514475 7.965598
H -1.266612 5.268078 8.201292
H -0.775139 7.338017 6.865138
H -2.524421 7.610264 6.627475
H -4.498701 6.883609 3.231375
H -4.220929 6.932170 4.986911
H 1.726435 9.948555 4.087804
H 2.720676 8.530659 3.686506
H -6.157177 9.269908 0.134264
H -5.729576 8.023083 1.340242
H -0.877677 5.159090 5.637852
H -2.643335 5.303706 5.430898
H -2.296244 6.382856 10.223127
H -1.113306 7.486735 9.445005
H -2.873855 7.744017 9.203017
H -0.977905 10.475678 0.635589
H -1.658394 11.416323 -0.713866
H -6.953836 6.262139 -0.087793
H -7.446492 7.557482 -1.203231
H -0.072864 9.121318 2.492642
H 0.914639 7.678672 2.133555
H -6.321836 5.964246 -7.781976
H -5.566152 7.324417 -6.886862
H -5.920964 5.786981 -6.037804
H 4.658469 6.666240 -8.697494
H 2.947264 6.137639 -8.788497
H 4.119203 5.176940 -7.839789
H -4.219297 4.594594 -7.555363
H -3.854399 6.153863 -8.335729
H -1.442844 6.299616 -7.657958
H -1.613493 4.610167 -7.089723
H -2.139558 10.011580 -4.815875
H -0.455055 10.372595 -4.367743
H -1.613603 7.562731 -4.273468
H 0.123977 7.799376 -4.038996
H 3.180639 8.070996 -7.182712
H 4.451635 7.211666 -6.281230
H 1.221414 5.172118 -7.187834
H 0.861808 6.926877 -7.354971
H -3.192437 7.053760 -5.999531
H -3.345200 5.385560 -5.375224
H 2.844907 5.520655 -5.448834
H 2.291180 7.172173 -5.083222
H -1.336290 8.532859 -6.692600
H 0.353100 8.907443 -6.277939
H -1.424752 12.234435 -5.775669
H -1.763453 11.076939 -7.106330
H -0.067985 11.446050 -6.648419
H -6.575446 -0.864377 -5.034904
H -7.230268 -0.356854 -6.608809
H -10.559291 5.244384 -4.188992
H -9.684014 5.343061 -2.637797
H -7.875495 4.216181 -8.793970
H -7.453793 2.594502 -9.392169
H -8.687321 -2.262936 -4.658303
H -7.656610 -2.784245 -6.011751
H -6.744483 3.609331 -6.597883
H -6.428870 1.927086 -7.050769
H -8.318771 4.983766 -5.384121
H -7.496658 4.981728 -3.793909
H -10.342399 3.757284 -9.276967
H -9.909636 2.129098 -9.894995
H -9.334859 3.597222 -10.755159
H -8.876059 1.558876 -7.560321
H -9.278702 3.188392 -6.966473
H -9.271039 0.657661 -5.349850
H -8.471174 0.198553 -3.812764
H -9.227579 -1.649328 -7.664894
H -10.302640 -1.164940 -6.310604
H -10.063662 -2.899447 -6.684465
H -8.792391 2.969754 -3.072737
H -9.669379 2.931648 -4.646736
H 2.060519 -9.271432 2.001030
H 1.390198 -8.322414 0.651506
H 6.277456 -9.597475 2.268990
H 4.629509 -10.176978 2.616462
H 3.857740 -7.773516 3.008848
H 5.496961 -7.182715 2.705476
H 6.507383 -10.845750 5.862585
H 5.542968 -11.639962 4.572283
H 7.204387 -11.057238 4.221995
H 9.995533 -7.195570 0.704253
H 9.495824 -5.799866 -0.306288
H 10.520683 -7.105913 -1.010863
H 0.099689 -8.450339 -4.963724
H -0.866975 -6.995981 -4.570047
H 2.666769 -8.539811 -2.831740
H 2.464377 -8.798193 -4.588159
H 5.212022 -9.316304 -3.287785
H 4.799999 -9.409278 -5.015034
H 7.288071 -9.119711 -4.708047
H 6.596561 -7.699529 -5.563745
H 7.021143 -7.579397 -3.825653
H 3.889293 -10.165527 0.445298
H 3.191463 -9.208061 -0.890209
H 8.161716 -7.397597 -1.800133
H 8.607371 -8.754504 -0.736668
H 9.348963 -7.331307 5.016355
H 8.472051 -7.432237 3.452606
H 10.252949 -7.652012 3.497140
H -2.838818 -9.296735 -1.253274
H -3.473283 -10.549125 -2.373492
H -1.835190 -10.727189 -1.661109
H -0.863585 -11.420446 0.805357
H -0.202460 -10.571596 2.242578
H -0.885436 -9.626213 0.878784
H 6.436729 -8.654919 4.610526
H 4.788343 -9.229903 4.955809
H 3.016318 -5.514515 -7.965735
H 1.266625 -5.267936 -8.201300
H 0.775045 -7.337877 -6.865184
H 2.524317 -7.610304 -6.627669
H 4.498892 -6.883758 -3.231694
H 4.221078 -6.932503 -4.987208
H -1.726329 -9.948483 -4.087565
H -2.720589 -8.530603 -3.686290
H 6.157119 -9.270136 -0.134289
H 5.729613 -8.023247 -1.340240
H 0.877877 -5.159027 -5.637793
H 2.643532 -5.303799 -5.431011
H 2.296020 -6.382736 -10.223245
H 1.113019 -7.486527 -9.445093
H 2.873557 -7.743992 -9.203224
H 0.977628 -10.475919 -0.635076
H 1.658524 -11.416365 0.714302
H 6.953951 -6.262420 0.087689
H 7.446410 -7.557678 1.203307
H 0.072999 -9.121080 -2.492491
H -0.914497 -7.678411 -2.133579
H 6.321959 -5.964446 7.781983
H 5.566145 -7.324559 6.886889
H 5.921040 -5.787149 6.037841
H -4.658346 -6.665974 8.697922
H -2.947132 -6.137423 8.788866
H -4.119050 -5.176742 7.840133
H 4.219536 -4.594641 7.555406
H 3.854550 -6.153848 8.335840
H 1.442988 -6.299466 7.658214
H 1.613663 -4.610067 7.089854
H 2.139470 -10.011670 4.816157
H 0.454934 -10.372597 4.368117
H 1.613647 -7.562776 4.273826
H -0.123910 -7.799415 4.039344
H -3.180573 -8.070847 7.183201
H -4.451567 -7.211558 6.281700
H -1.221316 -5.172012 7.188102
H -0.861717 -6.926749 7.355327
H 3.192457 -7.053726 5.999641
H 3.345258 -5.385528 5.375391
H -2.844835 -5.520651 5.449145
H -2.291129 -7.172188 5.083662
H 1.336385 -8.532900 6.692907
H -0.353038 -8.907400 6.278344
H 1.424606 -12.234481 5.775981
H 1.763446 -11.077009 7.106617
H 0.067937 -11.446028 6.648811
H 6.575676 0.864275 5.035215
H 7.230860 0.356659 6.608945
H 10.559309 -5.244787 4.188320
H 9.683717 -5.342984 2.637277
H 7.875955 -4.215987 8.794033
H 7.454345 -2.594233 9.392097
H 8.687310 2.262964 4.658219
H 7.656916 2.784113 6.011966
H 6.744824 -3.609333 6.597901
H 6.429253 -1.927071 7.050711
H 8.319289 -4.983830 5.384015
H 7.496611 -4.981694 3.794102
H 10.342898 -3.757119 9.276813
H 9.910232 -2.128847 9.894680
H 9.335473 -3.596851 10.755060
H 8.876467 -1.558821 7.560073
H 9.279065 -3.188383 6.966329
H 9.271402 -0.657682 5.349429
H 8.471125 -0.198484 3.812574
H 9.228415 1.649193 7.664623
H 10.303137 1.164965 6.310003
H 10.064153 2.899433 6.684051
H 8.792502 -2.969845 3.072532
H 9.669598 -2.931725 4.646475
H -3.190400 0.700701 8.744316
H -1.611052 0.689824 7.934355
H -6.666979 -4.124420 7.582025
H -6.547192 -5.200116 6.149337
H -5.507953 -3.739868 6.265678
H -8.837800 2.102755 3.775988
H -8.013100 1.735017 2.211562
H -8.790092 3.324769 2.449361
H -0.901341 2.726512 9.149390
H -2.551307 3.025348 9.744369
H -6.562852 1.622182 7.198633
H -5.354271 1.407760 8.485848
H -7.103548 3.887392 4.263758
H -6.304139 3.554704 2.698173
H -6.686432 1.394967 4.866297
H -5.654370 1.288413 3.411564
H -5.580113 -0.374325 5.936846
H -4.388742 -0.623155 7.221125
H -9.977445 -4.835763 2.935207
H -10.951025 -4.282178 1.550481
H -7.876975 -4.785521 1.468313
H -8.846771 -4.217688 0.099745
H -7.568127 -2.259825 6.136974
H -8.615659 -3.695531 6.040554
H -6.278259 -0.903058 8.965274

H -7.507975 -0.666826 7.702800
H -12.399552 -6.454698 1.702841
H -11.570797 -8.046577 1.643780
H -11.414342 -7.005960 3.098437
H -6.437610 -2.956634 3.954083
H -7.530951 -4.360652 3.868959
H -9.317254 -6.909638 1.647206
H -10.293740 -6.363210 0.263176
H -2.173007 2.833522 6.724845
H -3.731313 2.939221 7.602086
H -4.167679 3.130221 4.321142
H -5.211085 3.262265 5.776808
H -9.538662 -2.729510 3.850993
H -8.378281 -1.368209 3.852545
H -8.452984 1.330103 8.983674
H -7.216963 1.084938 10.261628
H -8.474304 -0.165000 9.978769
H -2.487114 0.962479 11.173211
H -0.873659 0.529348 10.531588
H -1.067856 2.000801 11.546043
H -11.570348 -0.512077 -1.726975
H -12.368756 -1.044968 -0.228518
H -10.276455 -2.325377 -0.740531
H -9.307017 -0.826436 -0.714629
H -9.816868 -0.480847 1.718318
H -10.834864 -1.953508 1.746645
H -11.510311 0.992781 0.999334
H -10.547779 1.480550 -0.432205
H -12.345895 1.495825 -0.500389
H 3.190343 -0.700694 -8.744520
H 1.611053 -0.689593 -7.934421
H 6.667322 4.124154 -7.582304
H 6.547825 5.200075 -6.149745
H 5.508287 3.740001 -6.265835
H 8.837981 -2.102475 -3.776424
H 8.013287 -1.735043 -2.211914
H 8.790411 -3.324679 -2.449999
H 0.901054 -2.726324 -9.149274
H 2.550933 -3.025259 -9.744462
H 6.562780 -1.622070 -7.198941
H 5.354156 -1.407670 -8.486120
H 7.103821 -3.887155 -4.264410
H 6.304484 -3.554767 -2.698721
H 6.686430 -1.394625 -4.866478
H 5.654437 -1.288493 -3.411664
H 5.580117 0.374413 -5.937121
H 4.388679 0.623284 -7.221340
H 9.977485 4.835550 -2.935463
H 10.951092 4.281938 -1.550756
H 7.877051 4.785381 -1.468513
H 8.846869 4.217561 -0.099948
H 7.568177 2.259574 -6.137043
H 8.616000 3.695096 -6.040849
H 6.278133 0.903158 -8.965591
H 7.507899 0.666911 -7.703162
H 12.399682 6.454421 -1.703201
H 11.570979 8.046330 -1.644085
H 11.414423 7.005731 -3.098750
H 6.437829 2.957011 -3.954209
H 7.531641 4.360691 -3.869284
H 9.317394 6.909451 -1.647428
H 10.293932 6.362991 -0.263437
H 2.172890 -2.833360 -6.724900
H 3.731165 -2.939147 -7.602202
H 4.167868 -3.130165 -4.321416
H 5.211199 -3.262042 -5.777162
H 9.538818 2.728970 -3.851089
H 8.378051 1.367973 -3.852656
H 8.452826 -1.330037 -8.984066
H 7.216781 -1.084835 -10.261992
H 8.474157 0.165076 -9.979149
H 2.486671 -0.962379 -11.173303
H 0.873290 -0.529197 -10.531521
H 1.067348 -2.000670 -11.545983
H 11.570604 0.512543 1.726968
H 12.368813 1.045181 0.228313
H 10.276326 2.325391 0.740413
H 9.307128 0.826299 0.714597
H 9.816843 0.480620 -1.718268
H 10.834858 1.953282 -1.746787
H 11.510485 -0.992954 -0.998985
H 10.548127 -1.480473 0.432754
H 12.346247 -1.495569 0.500784
H 1.031248 3.710781 10.461407
H 2.360150 3.596388 11.643105
H -0.283803 -3.332541 10.822218
H 0.918018 -2.761098 12.006159
H 2.073547 5.974951 10.852124
H 3.674185 5.351911 10.326580
H 2.324040 5.456601 9.145142
H 1.261821 1.203192 9.974214
H 2.587936 1.095262 11.166691
H 7.505067 -0.363117 10.398218
H 7.241734 1.393627 10.288495
H 4.938133 1.111944 11.291497
H 5.190805 -0.648299 11.387823
H 2.573210 2.897696 8.626006
H 3.914209 2.810422 9.797977
H 3.361603 -2.409559 9.727122
H 2.954441 -1.398266 11.151126
H 0.998439 -2.350952 8.929684
H 0.551517 -1.151529 10.153598
H 6.999315 1.523356 12.825068
H 8.581421 0.819024 12.350692
H 7.262476 -0.248698 12.937154
H 5.255463 0.988339 8.736451
H 5.423232 -0.772867 8.859210
H 2.656376 -4.342215 11.036762
H 1.485601 -4.860533 9.777699
H 1.175522 -5.233327 11.507269
H 6.729224 5.067599 5.767064
H 5.143149 4.284514 6.030169
H 7.199396 3.931513 3.630977
H 7.029796 4.198301 1.890787
H 5.556277 6.991189 4.555344
H 3.973485 6.207623 4.912297
H 4.473043 4.991677 0.207883
H 6.040504 5.845202 0.234458
H 9.589813 4.751702 3.742303
H 9.584978 4.940403 1.974667
H 5.737939 6.518280 -2.259928
H 4.248423 7.396005 -2.747334
H 4.158085 5.658745 -2.293559
H 7.700263 6.627537 2.160684
H 7.730784 6.452758 3.931714
H 11.418346 6.333419 3.015455
H 10.208161 7.404170 2.230205
H 10.211389 7.211978 4.013577
H 6.131292 7.834409 8.352684
H 7.333857 7.229300 7.164020
H 5.873239 8.112221 6.603820
H 4.396785 6.209269 7.443284
H 5.828551 5.373818 8.088604
H 5.257273 7.305935 2.137719
H 3.620142 6.583858 2.033387
H 3.302061 7.124448 -0.419413
H 4.849610 8.014580 -0.374025
H -1.031584 -3.710507 -10.461675
H -2.360723 -3.596197 -11.643108
H 0.283378 3.332590 -10.822642
H -0.918728 2.761280 -12.006363
H -2.073773 -5.974743 -10.852187
H -3.674371 -5.351837 -10.326366
H -2.324027 -5.456426 -9.145159
H -1.262248 -1.202944 -9.974508
H -2.588628 -1.095122 -11.166686
H -7.505519 0.363337 -10.397550
H -7.242190 -1.393410 -10.287885
H -4.938711 -1.111763 -11.291141
H -5.191352 0.648484 -11.387458
H -2.573218 -2.897509 -8.625963
H -3.914480 -2.810362 -9.797644
H -3.361859 2.409754 -9.726829
H -2.955031 1.398527 -11.150985
H -0.998521 2.350983 -8.929879
H -0.551928 1.151580 -10.153955
H -7.000055 -1.523101 -12.824494
H -8.582106 -0.818772 -12.349926
H -7.263223 0.248954 -12.936522
H -5.255716 -0.988145 -8.736065
H -5.423484 0.773071 -8.858803
H -2.656798 4.342451 -11.036483
H -1.485701 4.860622 -9.777648
H -1.175991 5.233502 -11.507272
H -6.729454 -5.067692 -5.766987
H -5.143407 -4.284503 -6.030000
H -7.199461 -3.931477 -3.630882
H -7.029858 -4.198178 -1.890679
H -5.556550 -6.991218 -4.555260
H -3.973743 -6.207731 -4.912207
H -4.472320 -4.991715 -0.207981
H -6.040132 -5.844571 -0.234149
H -9.589734 -4.751541 -3.742632
H -9.585299 -4.939977 -1.974974
H -5.737802 -6.517925 2.260016
H -4.248546 -7.396062 2.747398
H -4.157743 -5.658827 2.293650
H -7.700658 -6.627276 -2.160330
H -7.730844 -6.452824 -3.931387
H -11.418552 -6.333002 -3.015968
H -10.208635 -7.403744 -2.230304
H -10.211444 -7.211796 -4.013694
H -6.131287 -7.834349 -8.352700
H -7.333929 -7.229361 -7.164058
H -5.873283 -8.112220 -6.603845
H -4.396905 -6.209157 -7.443185
H -5.828690 -5.373754 -8.088508
H -5.257822 -7.305857 -2.137429
H -3.620469 -6.584281 -2.033564
H -3.302125 -7.124803 0.419502
H -4.849919 -8.014442 0.374100

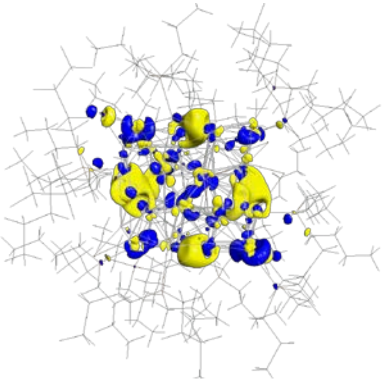

LUMO

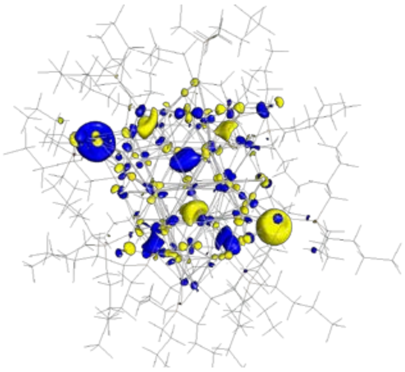

HOMO

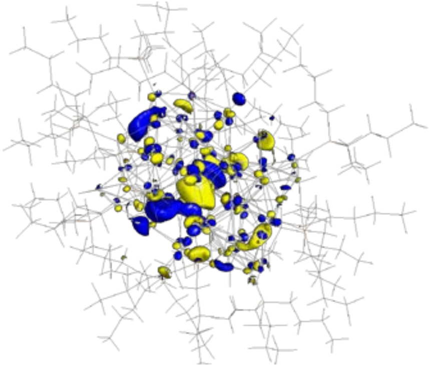

HOMO-1

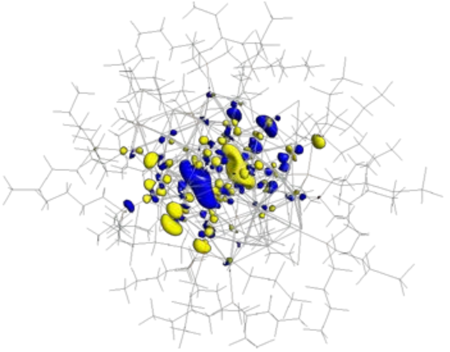

HOMO-2

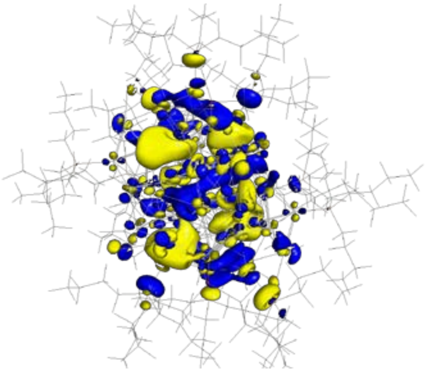

HOMO-3

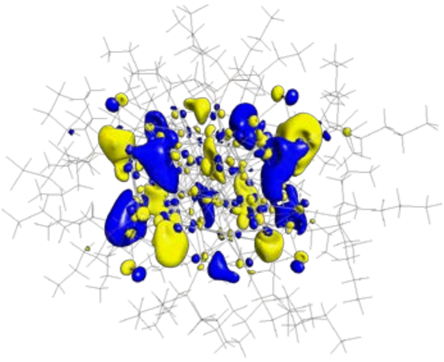

HOMO-4

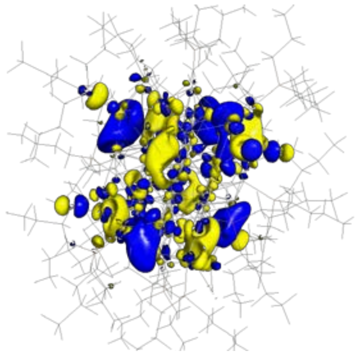

HOMO-5

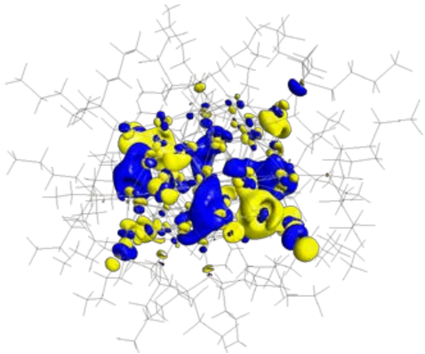

HOMO-6

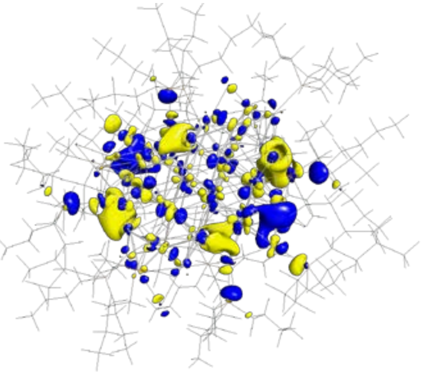

HOMO-7

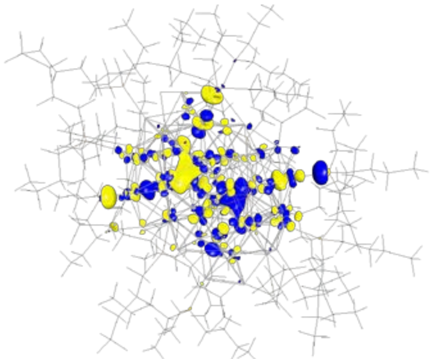

HOMO-8

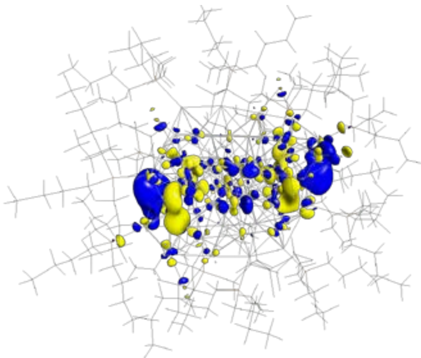

HOMO-9

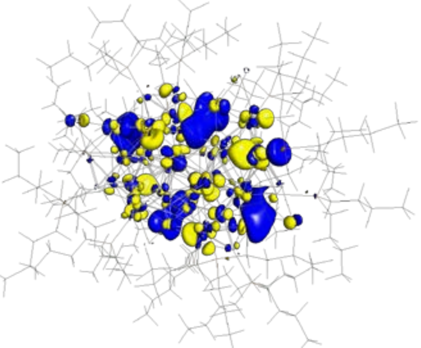

HOMO-10

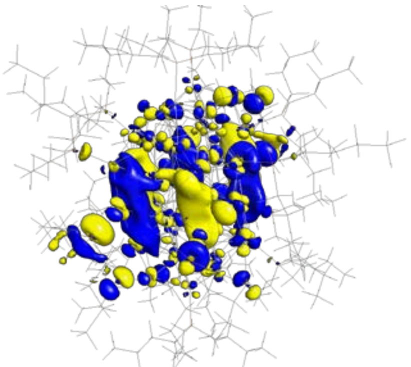

HOMO-11

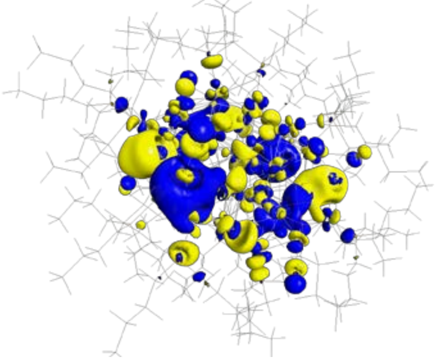

HOMO-12

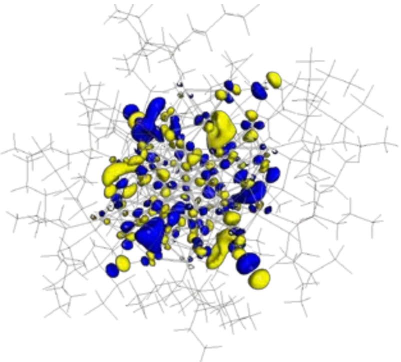

HOMO-13

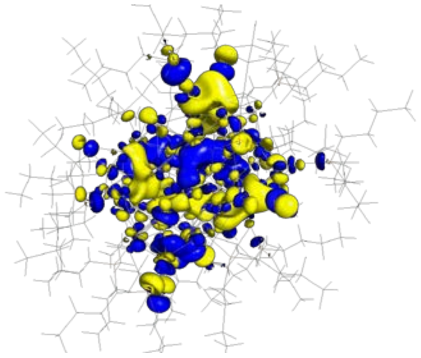

HOMO-14

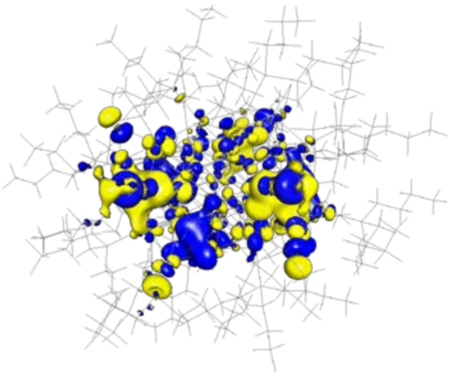

HOMO-15

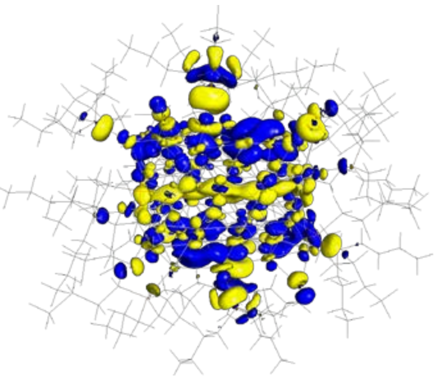

HOMO-16

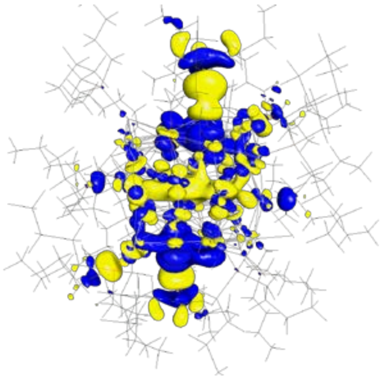

HOMO-18

Figure S10: Calculated cluster orbitals of **1**, displayed down to HOMO-18

6. References

[1] F. Hu, J.-J. Li, Z.-J. Guan, S.-F. Yuan, Q.-M. Wang, *Angew. Chem.* **2020**, 132, 5350 – 5353; *Angew. Chem. Int. Ed.* **2020**, *59*, 5312 – 5315.

[2] (a) G. M. Sheldrick, *Acta Crystallogr.* **2008**, *A64*, 112−122. (b) G. M. Sheldrick, *Acta Crystallogr., Sect. C: Struct. Chem.* **2015**, *C71*, 3−8.

[3] O. V. Dolomanov, L. J. Bourhis, R. J. Gildea, J. A. K. Howard, H. Puschmann, *J. Appl. Crystallogr.* **2009**, *42*, 339−341.

[4] A. L. Spek, *Acta Cryst.* **2009**, *D65*, 148 - 155.

[5] Turbomole: O. Treutler, R. Ahlrichs, *J. Chem. Phys.* **1995**, *102*, 346 – 354; BP86 functional: J. P. Perdew, *Phys. Rev. B* **1986**, *33*, 8822 – 88824; A. D. Becke, *Phys. Rev. A* **1988**, *38*, 3098 – 3100; RI-DFT: K. Eichkorn, O. Treutler, H. Öhm, M. Häser, R. Ahlrichs, *Chem. Phys. Lett.* **1995**, *240*, 283 – 290; SVP: A. Schäfer, H. Horn, R. Ahlrichs, *J. Chem. Phys.* **1992**, *97*, 2751 – 2577, TmoleX client: C. Steffen, K. Thomas, U. Huniar, A. Hellweg, O.Rubner, A. Schroer, *J. Comput. Chem.* **2010**, *31*, 2967 – 2970.
